# Promoting the Performance of Li–CO_2_ Batteries via Constructing Three-Dimensional Interconnected K^+^ Doped MnO_2_ Nanowires Networks

**DOI:** 10.3389/fchem.2021.670612

**Published:** 2021-04-15

**Authors:** Zhuolin Tang, Mengming Yuan, Huali Zhu, Guang Zeng, Jun Liu, Junfei Duan, Zhaoyong Chen

**Affiliations:** ^1^College of Materials Science and Engineering, Changsha University of Science and Technology, Changsha, China; ^2^School of Physics and Electronic Science, Changsha University of Science and Technology, Changsha, China

**Keywords:** Li-CO_2_ batteries, K^+^ doped MnO_2_ nanowires, interconnect networks, CO_2_ conversion, low overpotential

## Abstract

Nowadays, Li–CO_2_ batteries have attracted enormous interests due to their high energy density for integrated energy storage and conversion devices, superiorities of capturing and converting CO_2_. Nevertheless, the actual application of Li–CO_2_ batteries is hindered attributed to excessive overpotential and poor lifespan. In the past decades, catalysts have been employed in the Li–CO_2_ batteries and been demonstrated to reduce the decomposition potential of the as-formed Li_2_CO_3_ during charge process with high efficiency. However, as a representative of promising catalysts, the high costs of noble metals limit the further development, which gives rise to the exploration of catalysts with high efficiency and low cost. In this work, we prepared a K^+^ doped MnO_2_ nanowires networks with three-dimensional interconnections (3D KMO NWs) catalyst through a simple hydrothermal method. The interconnected 3D nanowires network catalysts could accelerate the Li ions diffusion, CO_2_ transfer and the decomposition of discharge products Li_2_CO_3_. It is found that high content of K^+^ doping can promote the diffusion of ions, electrons and CO_2_ in the MnO_2_ air cathode, and promote the octahedral effect of MnO_6_, stabilize the structure of MnO_2_ hosts, and improve the catalytic activity of CO_2_. Therefore, it shows a high total discharge capacity of 9,043 mAh g^−1^, a low overpotential of 1.25 V, and a longer cycle performance.

## Introduction

The excessive use of fossil resources makes the earth's carbon dioxide (CO_2_) flux unable to reach an effective balance, which has a huge impact on global warming, human health (Chu and Majumdar, 2012; Sanz-Perez et al., 2016). In recent years, continuous studies have been devoted to develop advanced CO_2_ capture and storage technologies for the reduction of CO_2_ emissions (Nguyen and Dinh, 2020). Among them, direct electrochemical reduction of CO_2_ with electron transfer is an effective solution for the capture and conversion of CO_2_ into sustainable energy (Khurram et al., 2018). Compared to other CO_2_ conversion techniques such as algae-based biofuel production (De Bhowmick et al., 2019), carbonate fixation (Jang et al., 2016), electrochemical conversion and energy storage devices (Appel et al., 2013), Li-CO_2_ battery has a higher discharge potential and specific energy density, so it is considered to be one of the most promising candidates (Xie et al., 2017).

In Li–CO_2_ battery, CO_2_ is served as working gas to provide and store energy sources by releasing and receiving electron during reduction and oxidation reaction process. This new energy device can not only capture CO_2_ but also convert CO_2_ to other pollution-free products, which can alleviate CO_2_ concentration in the environment (Li et al., 2016; Qiao et al., 2017). Besides, the theoretical specific energy of Li–CO_2_ battery can reach 1876 Wh Kg^−1^ based on the reaction of 4Li + 3CO_2_ → 2Li_2_CO_3_ + C (Liu et al., 2014; Zhang et al., 2015b; Li et al., 2016). Despite its huge potential, it still faces many difficulties. Firstly, it requires high energy to active the CO_2_ due to the thermodynamic stability of CO_2_, which results in the sluggish CO_2_ reduction reaction (CO_2_RR) kinetics (Pipes et al., 2019; Qiao et al., 2019). Secondly, the main discharge product Li_2_CO_3_ produced by the reaction is a wide-band gap insulating material, which shows a higher decomposition potential (>4.3 V vs. Li^+^/Li) during charging (Garcia-Lastra et al., 2013; Ling et al., 2014; Liu et al., 2017). Therefore, this discharge product leads to a large overpotential, cycle instability, and poor rate performance, ultimately battery failure (Lim et al., 2013; Ling et al., 2014; Li et al., 2017; Xie et al., 2017; Yin et al., 2017).

In order to solve these problems, researchers have proven that good catalysts can promote the decomposition of Li_2_CO_3_ and improve the performance of Li–CO_2_ batteries. Nanocarbon materials [carbon nanotubes (Zhang et al., 2015a), graphene (Zhang et al., 2015b), KB (Wang et al., 2017) etc.] and heteroatom-doped carbon materials (Qie et al., 2017), precious metals (Yang et al., 2017; Wang et al., 2018), transition metals (Zhang et al., 2018b, 2019) and their oxides (Zhang et al., 2018a; Lu et al., 2019), carbides (Hou et al., 2017; Zhou et al., 2018a) etc. are investigated. These catalysts can not only effectively reduce the decomposition potential (<4 V) of Li_2_CO_3_, promote the kinetics and thermodynamics of CO_2_ reduction reaction (CO_2_RR) and CO_2_ evolution reaction (CO_2_ER), but also improve the cycling stability of the battery (Qiao et al., 2017; Liu et al., 2019a), However, the rechargeability of Li–CO_2_ battery is still far from the level of practical application because of low cycle performance, high charge potential and large overpotential (Hu et al., 2019; Liu et al., 2019a), and it is important to further design high efficient and low-cost catalysts (Liu et al., 2019a). Mn-based oxides are one of the promising catalysts because of their various crystal structures, high activity and low-cost prices (Chen et al., 2012; Zhang et al., 2015).

**Scheme 1:**
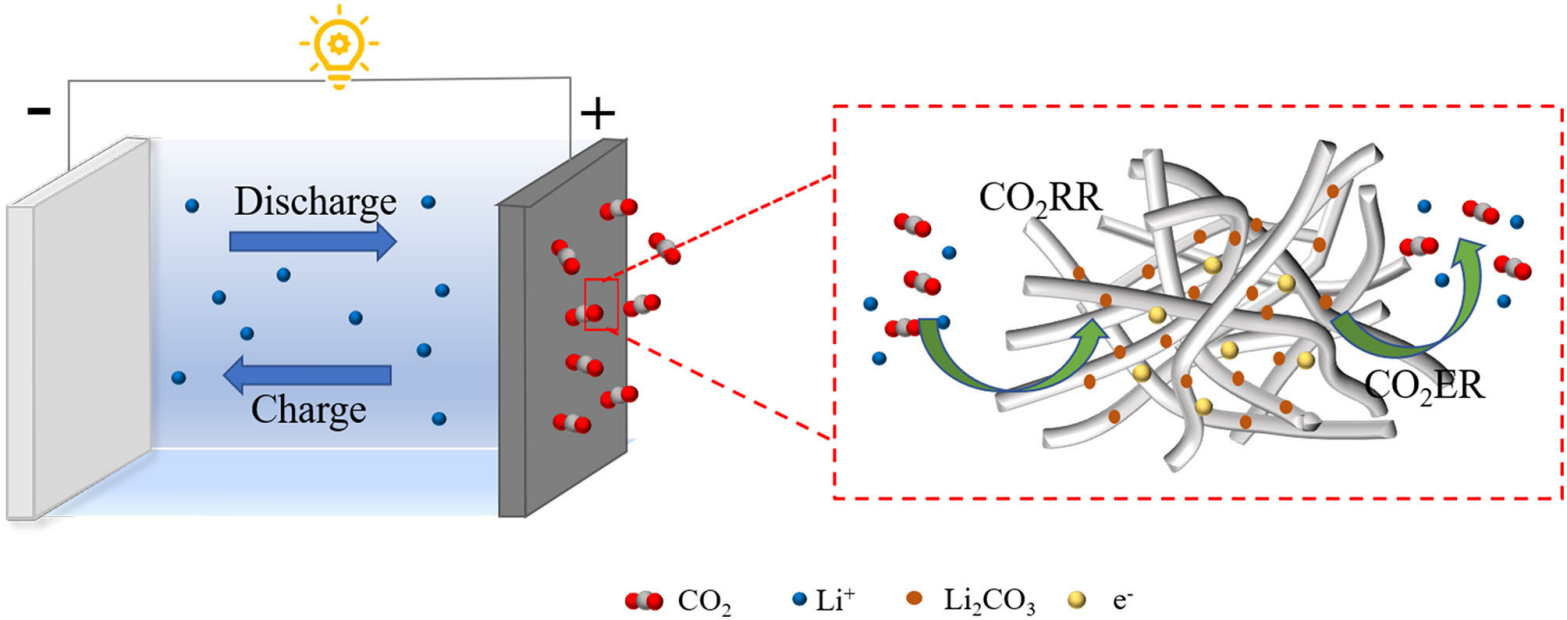
Schematic illustration of the proposed catalytic mechanism based on the 3D KMO NWs cathode catalyst.

Because of its rich crystal structure, adjustable valence and unique MnO_6_ octahedral effect, MnO_2_ is widely used in the field of catalysis (Liu et al., 2018). However, the MnO_2_ cathode will undergo severe structural degradation during the cycle, resulting in a decrease in cycle performance (Zhang et al., 2017). During the synthesis process, the tunnel structure of MnO_2_ is inserted into cations (such as K^+^, Ba^2+^) to stabilize the structure between the guest ion and the host atom (Zhai et al., 2011; Yuan et al., 2015). In addition, the intercalation of cations can also enhance the diffusion of ions in the MnO_2_ structure, thereby increasing the electronic conductivity to enhance its catalytic effect (Hao et al., 2019). Therefore, in this work, we synthesized K^+^ doped MnO_2_ nanowires networks with three-dimensional interconnections (3D KMO NWs) by a simple hydrothermal method. It should be noted that KMO nanowires with 3D interconnected structure can facilitate charge transfer and boost energy storage. As expected, the as-prepared 3D KMO NWs catalyst with interconnected network could promote the transfer of CO_2_ and the decomposition of discharge products effectively (Scheme 1). Meanwhile, K^+^ can effectively stabilize Mn-based materials, and the high content of K^+^ can promote the octahedral effect of MnO_6_ and the diffusion of ions, thereby enhancing the catalytic activity of main MnO_2_ hosts for CO_2_ER and CO_2_RR. As a result, which exhibit an improved specific discharge capacity of 9043.0 mAh g^−1^ with decreased overpotential and improved cycling stability.

## Experimental Section

### Materials Preparation

3D KMO NWs were prepared by a simple hydrothermal method. Typically, 20 mmol KMnO_4_ and 60 mmol urea were dissolved in 80 mL deionized water and stirred for 30 min. Afterwards, the obtained solution was transferred to a 100 mL Teflon-lined autoclave, then sealed and heated for 12 h at 120°C. Subsequently, 3D KMO NWs were collected after filtration and drying at 80°C for 12 h. In addition, two other samples were also prepared under different ration of KMnO_4_ to urea.

### Materials Characterizations

X-ray diffraction (XRD) patterns of as-prepared samples were investigated using a Bruker D8 X-ray diffractometer with a scanning rate of 5° min^−1^. The microstructure morphologies were observed using scanning electron microscopy (SEM, JSM-7900F) and transmission electron microscopy (TEM, TECNAI G2 F20). N_2_ adsorption/desorption isotherms were obtained by Novatouch LX2. The element analysis of the sample is tested by X-ray photoelectron spectroscopy (XPS, ESCALAB 250Xi). Raman spectra were recorded using a Renishaw in-Via confocal Raman spectrometer.

### Electrochemical Measurements

To assess the energy storage properties, CR2032 coin-type battery was assembled on the condition of H_2_O and O_2_ lower than 0.01 ppm with lithium foil, GD/F Whatman glass fibers and 1 M LiTFSI in TEGDME organic solution as counter electrode, separators and electrolyte, respectively. To prepare an air electrode, 60 wt% of 3D KMO NWs, 30 wt% of KB and 10 wt% PVDF were mixed and made into slurry with NMP as solvent and cast on carbon paper. After drying at 80°C under vacuum, the electrodes were cut into disks with a diameter of 14 mm. Before testing, the as-assembled batteries were aged for 12 h in high-purity CO_2_ atmosphere. The cyclic voltammetry (CV) test was performed on a CHI660E electrochemical workstation, the scanning range is 2.0–4.5 V and the scanning rate is 0.2 mV s^−1^. Electrochemical impedance spectroscopy (EIS) curves were obtained in the frequency range of 0.01–100k Hz. Galvanostatic discharge and charge measurements were performed using a Shenzhen Neware BTS 3000 battery test system. For better comparison, bare electrode comprised of 90 wt% KB and 10 wt% PVDF was prepared in the same manner.

## Results and Discussion

**Figure 1:**
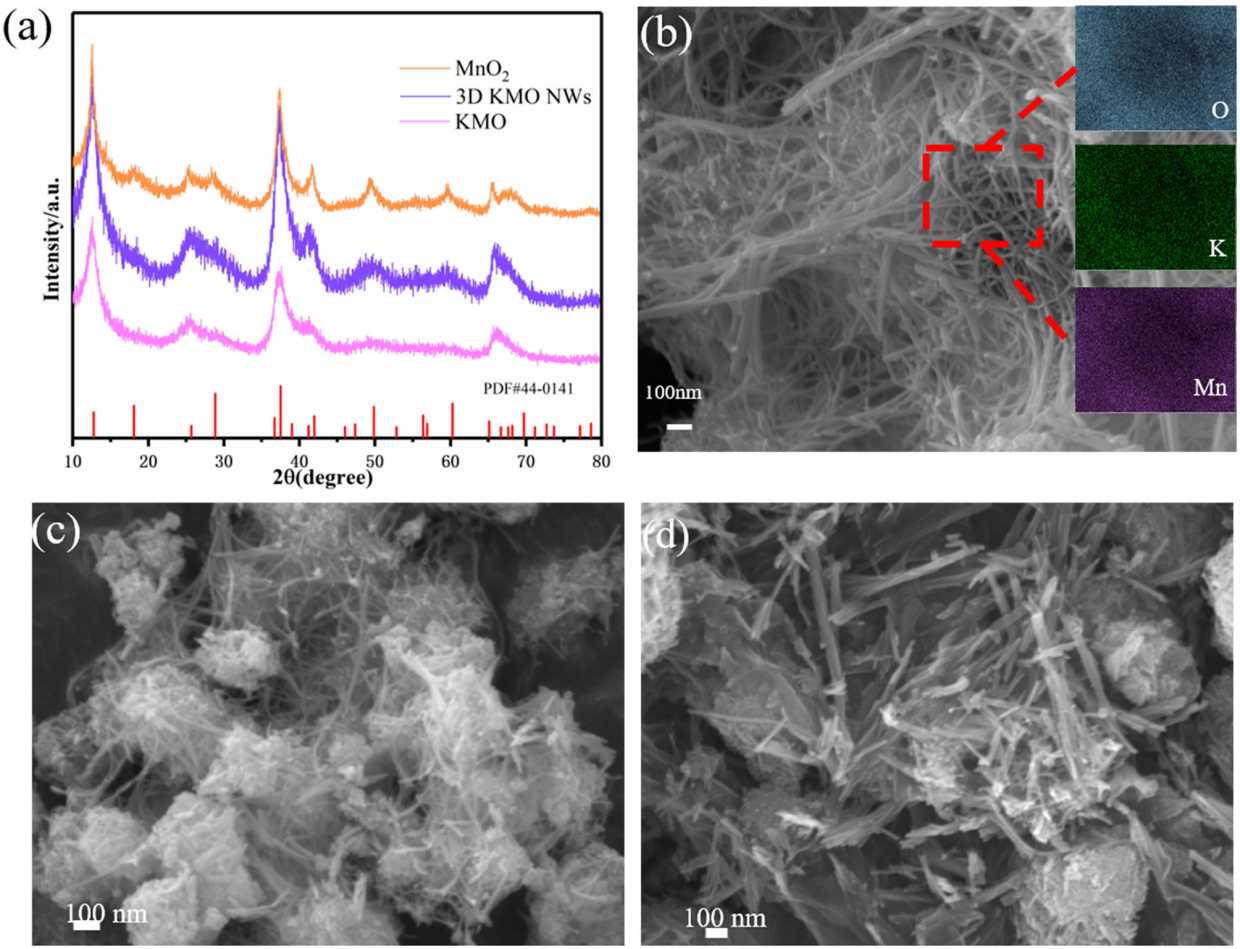
**(a)** XRD pattern of 3D KMO NWs, KMO and MnO_2_. **(b)** SEM images of 3D KMO NWs, and the inset of **(b)** is the element mapping of 3D KMO NWs. SEM images of KMO **(c)** and α-MnO_2_ **(d)**, respectively.

Typically, KMnO_4_ and urea solution were mixed with a specific molar ratio and afterwards underwent a facile hydrothermal process. During the hydrothermal process, urea is used to reduce KMnO_4_ to MnO_2_. As shown in Figure 1a, the XRD results of the products with different KMnO_4_ and urea ratios show that the peak strength of MnO_2_ gradually increases with the increase of urea concentration, and the prepared samples can be indexed as α-MnO_2_ (PDF # 44-0141). This may be due to the intercalation of K^+^ into MnO_2_, which makes the peak strength of KMO lower than that of α-MnO_2_, leading to the appearance of some peaks is not very obvious. Afterwards, the two samples were named as KMO and α-MnO_2_, respectively. Through the inductively coupled plasma optical emission spectrometry (ICP-OES) analysis, the chemical composition of KMO, 3D KMO NWs and α-MnO_2_ (the ratio of K and Mn) was determined, which were K_0.13_MnO_2_, K_0.18_MnO_2_, and K_0.08_MnO_2_, respectively. About 1.5 K^+^ in per 8 Mn is embedded in the main channel of MnO_2_. In theory, there is 2 K^+^ in per 8 Mn, which shows that K^+^ is approximately filled in the main channel of MnO_2_ (Liu et al., 2019b). The morphologies and structures of as-prepared 3D KMO NWs were observed by scanning electron microscopy (SEM) and transmission electron microscopy (TEM). The SEM images in Figure 1b clearly presented a 3D interconnected networks comprised of considerable KMO nanowires with 10~20 nm in diameter and several micrometers in length. It also can be seen from the result of EDX that K, Mn and O are evenly distributed in the sample. On the one hand, the as-prepared nanowires with high aspect ratios should account for high surface area, which contributed to shortening transfer paths and exposing more active sites for facilitate the reversible conversion of formed Li_2_CO_3_. On the other hand, the 1D nanowire structure could direct the electron transport axially owing to their inherent features, which lead to accelerate the reaction kinetics and boost the electrochemical performance. To make things better, apart from further promoting the charge transfer, the interconnected networks could improve the penetration of electrolyte and decrease transfer barriers (Mai et al., 2014). Through KMO (Figure 1c), the production of nanowires can still be seen, but there are still many particles that have not grown into nanowires clustered together, and the α-MnO_2_ are nanorods (Figure 1d).

**Figure 2:**
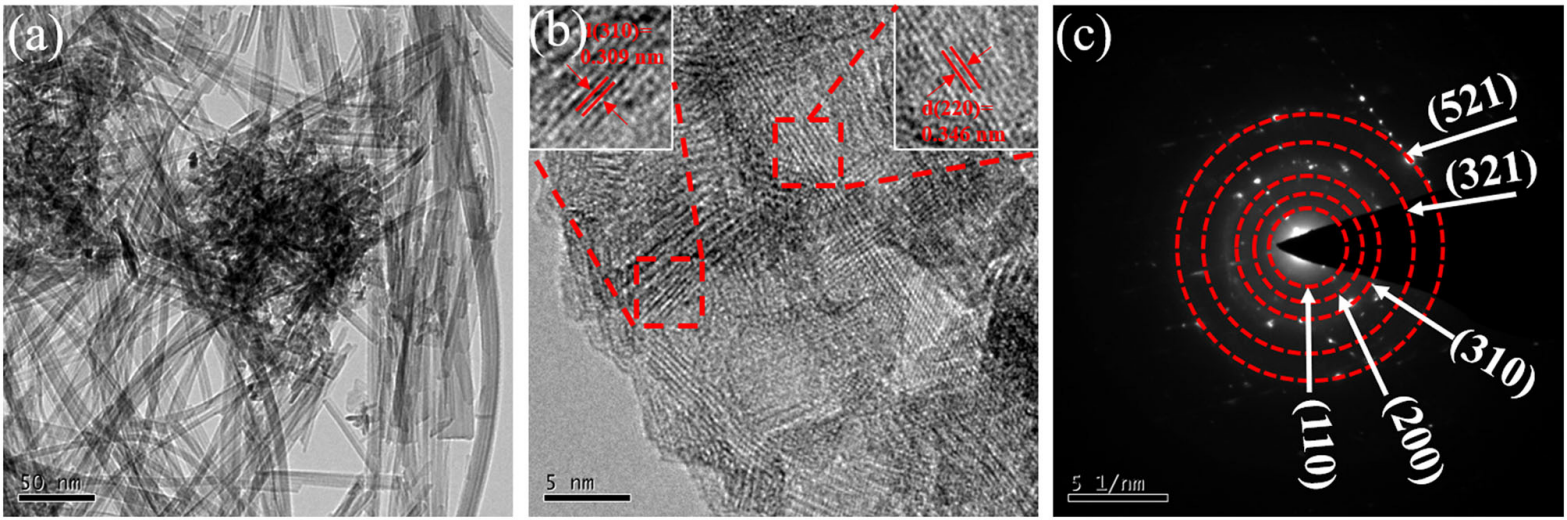
**(a)** TEM images. **(b)** HRTEM images, and **(c)** SAED pattern of 3D KMO NWs.

While in TEM images, as shown in Figure 2a, the 3D interconnected KMO networks obviously exhibited nanowires-like structure with interspaces, further enhancing the penetration of electrolyte and promoting electrochemical performance. The high-resolution TEM (HRTEM) images in Figure 2b show vast well-resolved lattice fringes, confirming the excellent crystallinity. The insets displayed typical lattice spacing of 0.346 nm and 0.309 nm, which corresponded to the (220) plane and (310) plane of α-MnO_2_, respectively. Moreover, five diffraction rings could be observed through the selected area electron diffraction (SAED) pattern (Figure 2c), confirming a polycrystalline feature. Additionally, the diffraction rings could be well-indexed to (110), (200), (310), (321), and (521) planes of α-MnO_2_, which corresponds to the results of XRD.

**Figure 3:**
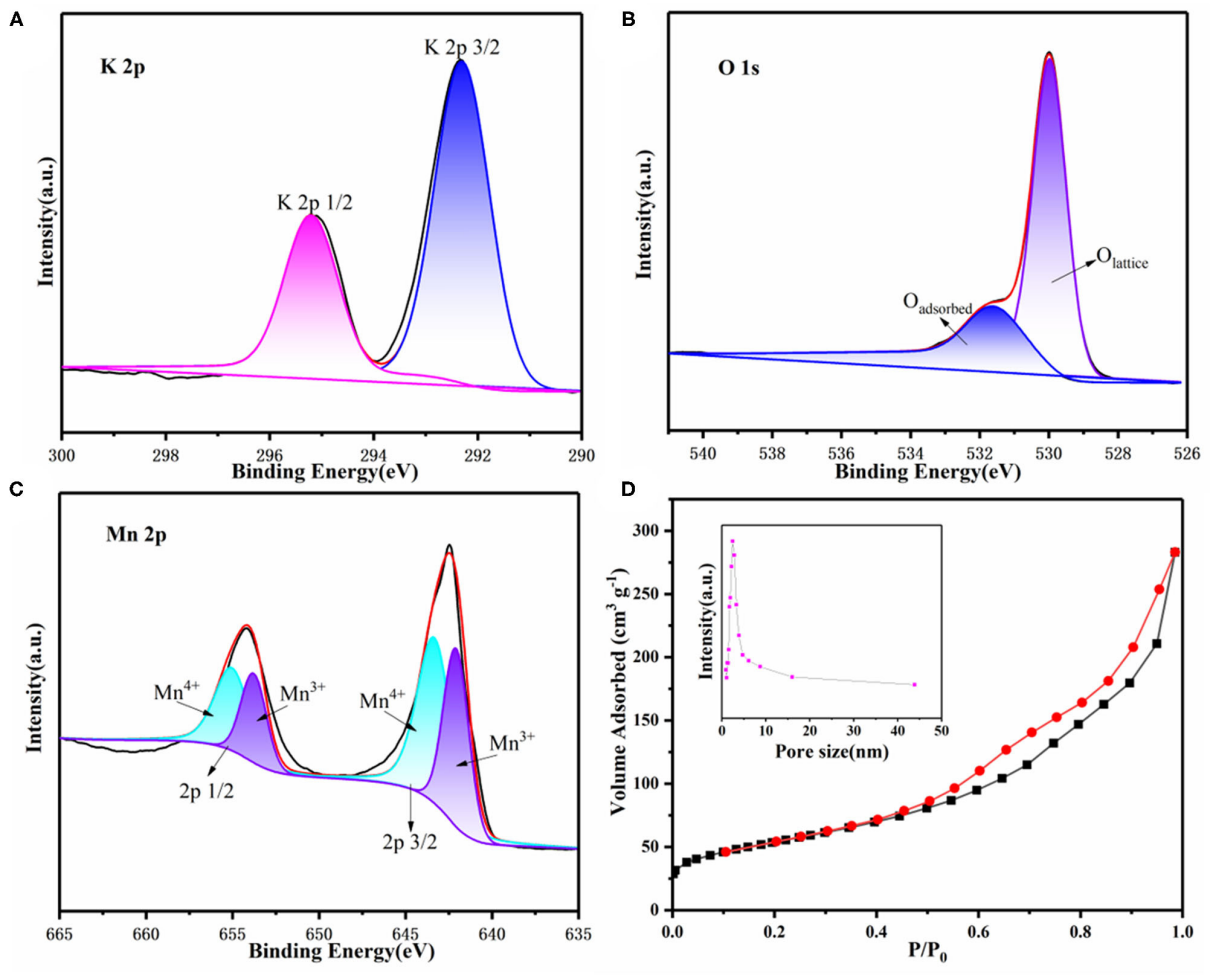
XPS survey of **(A)** K 2p, **(B)** O 1s and **(C)** Mn 2p and **(D)** N_2_ adsorption/desorption isotherm curves of 3D KMO NWs. The inset of (d) is the corresponding pore distribution of 3D KMO NWs.

To further demonstrate the composition of as-prepared samples, X-ray photoelectron spectroscopy (XPS) was performed as well. As shown in Supplementary Figure 1, the XPS survey of 3D KMO NWs exhibited K, Mn, O and C peaks. As shown in Figure 3A, theses peaks at 292.3 eV and 295.2 eV can be contributed to K (Qin et al., 2019). From Figure 3B, O 1s has two peaks at 529.8 eV and 531.7 eV, which are represented as lattice oxygen and adsorbed oxygen (Lee et al., 2001). In addition, Mn^3+^ and Mn^4+^ correspond to 642.3 eV and 643.5 eV, respectively (Figure 3C). The ratio of Mn^4+^ is higher than of Mn^3+^, this means that the presence of K^+^ could induce the charge-ordering of Mn^4+^ and Mn^3+^, promoting the electron transport of α-MnO_2_ (Liu et al., 2019b). N_2_ adsorption/desorption isotherms was recorded to further show 3D KMO NWs porous feature. Figure 3D shows a typical Type IV isotherm of 3D KMO NWs. Calculated by Brunauer-Emmett-Teller (BET) method, the specific surface area of 3D KMO NWs is 193.1 m^2^ g^−1^, therefore, the high specific surface area can also maximize the deposition of discharge products. The surface areas of KMO and α-MnO_2_ are smaller than those of 3D KMO NWs, which are 174.3 m^2^ g^−1^and 163.09 m^2^ g^−1^ (Supplementary Figure 2), respectively. Additionally, the pore size distribution of 3D KMO NWs is about 2-10 nm (inset of Figure 3D), which demonstrates that its porous structure is mainly contributed by mesopores. These pores combined 3D porous networks could not only promote the transmission of CO_2_ ions and electrolyte, but also provide enough space to deposit discharge products.

**Figure 4:**
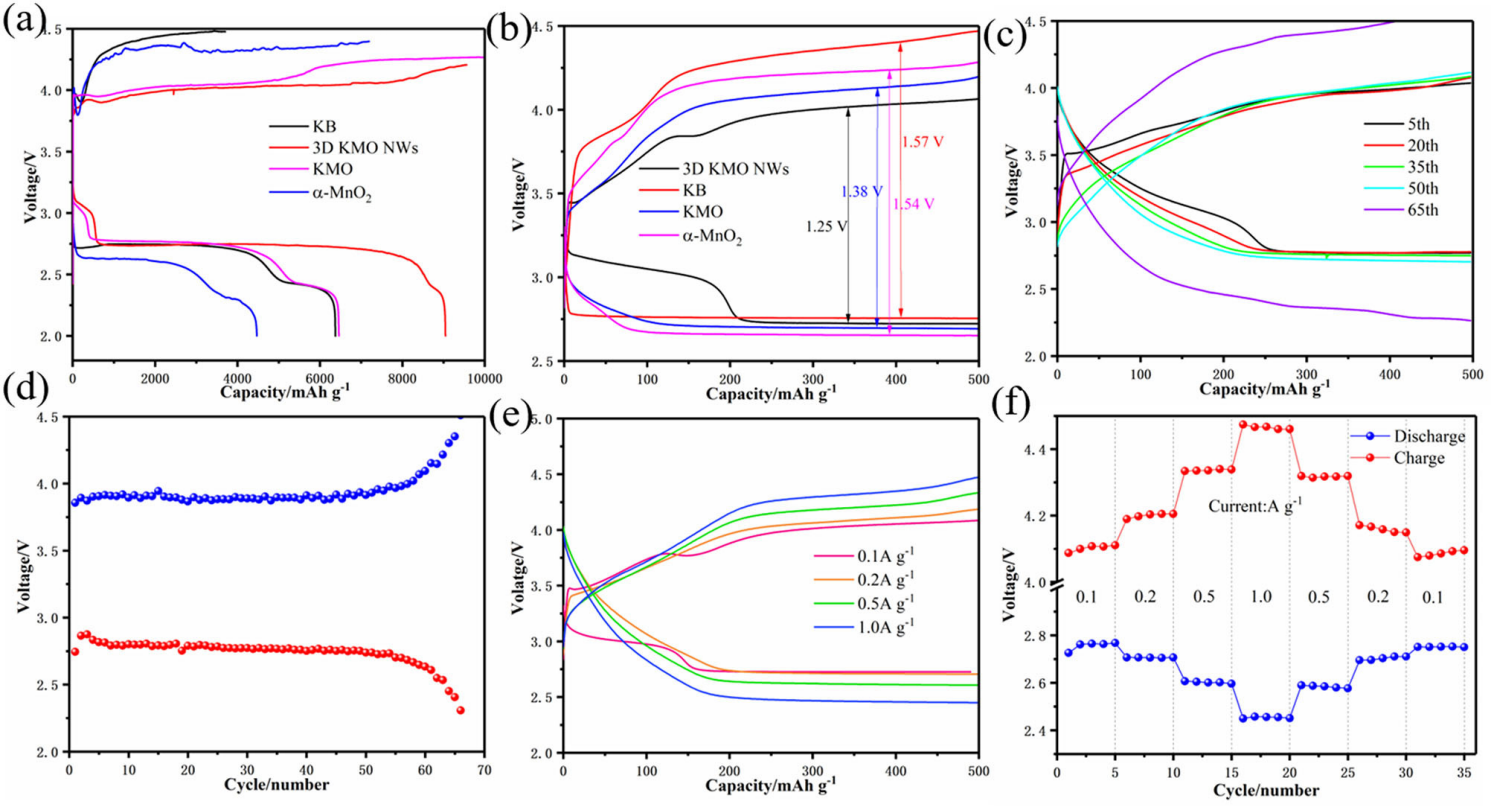
**(a)** the full discharge-charge voltage curves at a current density of 100 mA g^−1^, **(b)** the comparison of polarization at the first cycle, discharge-charge profiles of 3D KMO NWs, KMO, α-MnO_2_ and KB, **(c)** the discharge-charge voltage curves within a limiting capacity of 500 mAh g^−1^ at a current density of 100 mA g^−1^ of 3D KMO NWs. **(d)** the relationship between the median voltage of 3D KMO NWs and the number of cycles. **(e)** Comparison of cycle curves of Li–CO_2_ batteries with 3D KMO NWs at different current densities. **(f)** Rate performance of Li–CO_2_ battery with 3D KMO NWs cathode at different current densities.

In order to study the influence of 3D KMO NWs on CO_2_ER and CO_2_RR, Li–CO_2_ batteries were assembled and tested in a CO_2_ atmosphere. Firstly, we tested the cyclic voltammogram (CV) to study the electrochemical activity of 3D KMO NWs in Li–CO_2_ batteries with a voltage range of 2.0-4.5 V. Compared with KMO and α-MnO_2_ (Supplementary Figure 3b), the oxidation peak area of 3D KMO NWs is larger than both of them, and it is also compared with commercial KB (Supplementary Figure 3a). The curve shows that the cathode peak area of 3D KMO NWs is larger than that of pure commercial KB, and the potential of the anode peak of 3D KMO NWs cathode is higher than that of pure commercial KB cathode. In addition, the anode current of 3D KMO NWs cathode is higher than that of KB. The results show that the 3D KMO NWs/KB composite material has enhanced activity for discharge products (mainly about Li_2_CO_3_) decomposition. Secondly, to further prove the catalytic performance of 3D KMO NWs, the first cycle curves of Li–CO_2_ batteries with the 3D KMO NWs/KB, KMO, α-MnO_2_ and KB electrodes are shown in Figure 4a. The 3D KMO NWs/KB composite cathode exhibited the higher discharge capacity of 9,043 mAh g^−1^ (Liu et al., 2020). Even during the subsequent charging process, its terminal charging voltage is still lower than 4.3 V. The discharge capacities of KMO and α-MnO_2_ are 6,460 mAh g^−1^ and 4,468 mAh g^−1^, respectively, which are lower than 3D KMO NWs. In addition, the overpotential of the first cycle of 3D KMO NWs composite air cathode is ~1.25 V (Figure 4b), which is lower than that of pure commercial KB (1.57 V), KMO (1.38 V) and α-MnO_2_ (1.54 V). This indicates that α-MnO_2_ and KMO with lower K^+^ content have lower capacity in Li–CO_2_ batteries and higher overpotential. On the contrary, 3D KMO NWs with high K^+^ content shows good electrochemical performance. The increase in discharge capacity and the decrease in overpotential can be attributed to the large specific surface area and pore size of 3D KMO NWs, so that the discharge products can be deposited on the 3D KMO NWs to the greatest extent. On the other hand, high content of K^+^ can enhance the diffusion of ions and electrons in the MnO_2_ main channel, accelerate the transmission of ions and electrons, and provide more catalytic activity to promote the reaction kinetics of CO_2_ER and CO_2_RR.

The Li–CO_2_ batteries with the 3D KMO NWs/KB composite materials were evaluate their cycle performance by galvanostatic discharge/charge test at a current density of 100 mA g^−1^ with a cut-off capacity of 500 mAh g^−1^. Compared with others materials as cathodes, 3D KMO NWs/KB electrode showed about 64 cycles as shown in Figure 4c. As shown in Supplementary Figure 4, KMO can only cycle 33 times, while α-MnO_2_ can only cycle 31 times (Supplementary Figure 5). And also evaluating the pure commercial KB can only cycle 13 times in the voltage range of 2.0–4.5 V, and after 13 cycles (Supplementary Figure 6), the charging potential has exceeded 4.5 V, indicating that pure commercial KB cannot effectively decompose the discharge product Li_2_CO_3_.It is exhibited a good cycle performance with 3D KMO NWs/KB electrode. Through the 3D KMO NWs discharge and charge median voltage (Figure 4d), it is found that the overpotential has been stable at about 1.15V before 55 cycles. After 55 cycles, the overpotential increased rapidly, which may be due to the complete oxidation of the Li anode, finally caused the battery to fail (Shui et al., 2013; Li et al., 2019). The overpotential of the median voltage is lower than the overpotential of the first cycle. This is mainly due to the fact that after one cycle of activation of the battery, electrolyte and gas will completely infiltrate, making the electrons transmission easier. The electrochemical results show that the high content of K^+^ doping in the MnO_2_ hosts can stabilize the structure of MnO_2_, so that it has more stable cycle performance. Compared with the other two samples, the more K^+^ embedded in the main MnO_2_ hosts, the better the promotion of the octahedral effect of Mn-O, thereby enhancing the catalytic activity of the main MnO_2_ hosts for CO_2_ER and CO_2_RR.

Through the discharge-charge curves under different current densities (Figure 4e), the discharge platform of the battery is 2.72 V at a current density of 0.1 A g^−1^. After a cycle, the discharge platform of the battery has improved and stabilized at 2.76 V, while at different current densities of 0.2, 0.5, and 1.0 A g^−1^, their discharge platforms are 2.71, 2.61, and 2.46 V, respectively. The discharge platform is higher than 2.4 V at a large current density of 1.0 A g^−1^, indicating 3D KMO NWs has a good rate performance. In addition, the rate performance of Li–CO_2_ battery with 3D KMO NWs/KB cathode was studied by discharging/charging five cycles with a cut-off capacity of 500 mAh g^−1^ under different current densities. As shown in Figure 4f, even at a higher current density (1.0 A g^−1^), the charge potential of the 3D KMO NWs cathode is only 4.47 V, which is still lower than the set voltage range, and the discharge potential is also more than 2.4 V. In addition, after 30 cycles of different current densities, the charge potential returned to the previous 4.07 V, and the discharge potential remained the same as before. This shows that the Li–CO_2_ battery with 3D KMO NWs cathode has good reversibility and stability after high current density. Therefore, 3D KMO NWs catalyst can not only improve the reaction kinetics of CO_2_ER and CO_2_RR, but also effectively improve the electrochemical performance of Li–CO_2_ batteries (Table 1). The electrochemical reaction mechanism of Li–CO_2_ battery is still unclear, but the research on the above-mentioned electrochemical performance partially proves that α-MnO_2_ doped with different K^+^ content has a greater influence on the electrochemical reaction of Li–CO_2_. The high content of K^+^ doping can not only effectively increase the ion and electron diffusion in the tunnel of the MnO_2_ hosts, promote the octahedral effect of Mn-O, but also stabilize the structure of α-MnO_2_, which can improve the catalytic activity of CO_2_ and make it have more stable electrochemical performance.

**Table 1:** Comparison and summary of recent literatures on electrochemical performance of Li-CO_2_ batteries with different cathodes.

**Cathode**	**Current density (mA g^**−1**^)**	**Discharge capacity (mAh g^**−1**^)**	**Limited capacity (mAh g^**−1**^)**	**Cycle number**	**Overpotential(V)**	**References**
Ketjen black	50	1,620	1,000	9	-	Zhang et al., 2015b
Cu_2_O-Rd	100	6,235	500	50	1.9	Jena et al., 2020
Cu_2_O-Cb	100	5,014	500	45	2.2	Jena et al., 2020
Cu_2_O-Oh	100	3,314	500	45	2.4	Jena et al., 2020
Mn_3_O_4_	100	14,281	1,000	29	1.33	Liu et al., 2020
Ir/CNF	100	18,813	1,000	27	1.38	Wang et al., 2018
MnO@NC-G	50	25,021	1,000	15	0.88	Li et al., 2019
Ru/CNT	100	2,882	500	36	1.11	Chen et al., 2020
P-Mn_2_O_3_	50	9,434	1,000	50	1.40	Ma et al., 2018
CNT@RuO_2_	100	-	500	30	1.51	Bie et al., 2019
α-MnO_2_	100	4,468	500	31	1.54	This work
KMO	100	6,460	500	33	1.38	This work
3D KMO NWs	100	9,043	500	64	1.25	This work

**Figure 5:**
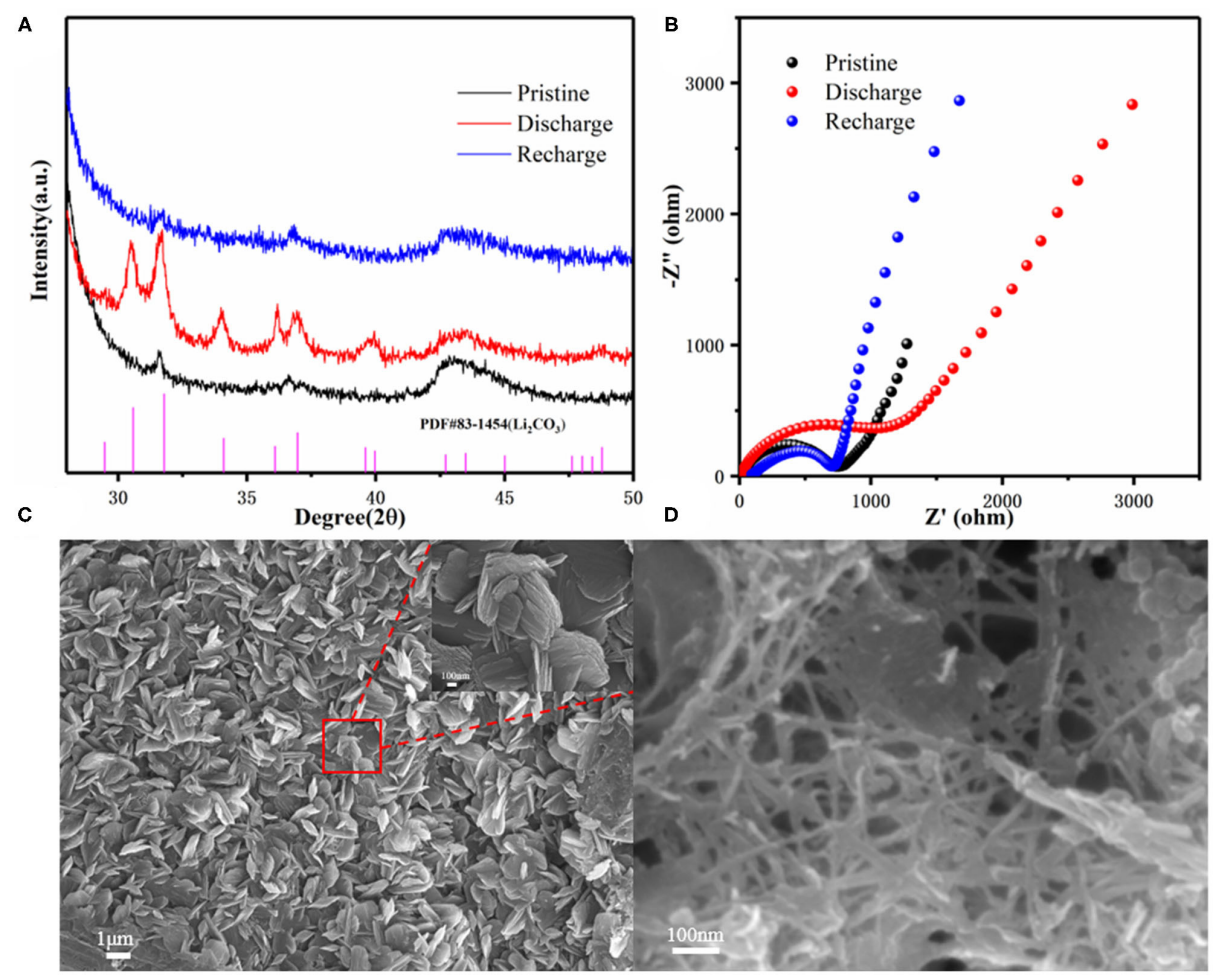
Li–CO_2_ battery **(A)** XRD of pristine, first discharge and first charge, **(B)** EIS of pristine first discharge and first charge, **(C)** SEM image after first discharge, inset is a partial enlarged image. **(D)** SEM image after first charging.

To further understand the reversible reduction and evolution of CO_2_ on the 3D KMO NWs cathode, we further characterized the formation and decomposition of discharge products in the electrochemical process through *ex-situ* XRD, EIS, XPS, and Raman. As shown in Figure 5A, there is no obvious XRD peak over 28° of 2θ displayed on the pristine 3D KMO NWs electrode except for the peak of carbon paper. The extra peaks in the XRD curve of the discharge electrode are all attributed to Li_2_CO_3_ (PDF#83-1454), and the disappearance of these peaks after the recharging process is attributed to the high reversibility of the efficient cathode catalyst. This is consistent with previous reports (Zhou et al., 2018b; Wang et al., 2019). Figure 5B shows the electrochemical impedance spectroscopy (EIS) spectra of the Li–CO_2_ batteries with 3D KMO NWs cathode. Compared with electrode of different electrochemical states, due to the formation of insulating Li_2_CO_3_ during the discharge process, the EIS pattern displayed a significantly larger semicircle by the discharge product, which increased the interface and charge transfer impedance (Qie et al., 2017). The subsequent recharging process, interface impedance resistance recovered similar to the EIS spectrum of the pristine electrode, indicating that the Li_2_CO_3_ had been efficiently decomposed during the charging process. This is corresponded to the XRD results (Hu et al., 2020).

In order to further clarify the discharging/charging processes of Li–CO_2_ battery. The 3D KMO NWs cathode after the discharging and charging processes were characterized by SEM. Figure 5C shows the discharging cathode, the thin plate-shaped discharge product Li_2_CO_3_ (≈300 nm) gradually deposited, aggregated and finally completely covered the electrode surface (Xing et al., 2018; Guo et al., 2019). After recharging, the discharge products disappear completely, and only 3D KMO NWs can be observed in Figure 5D. Therefore, it can be confirmed that the 3D interconnected 3D KMO NWs have good catalytic performance for the decomposition of Li_2_CO_3_.

**Figure 6:**
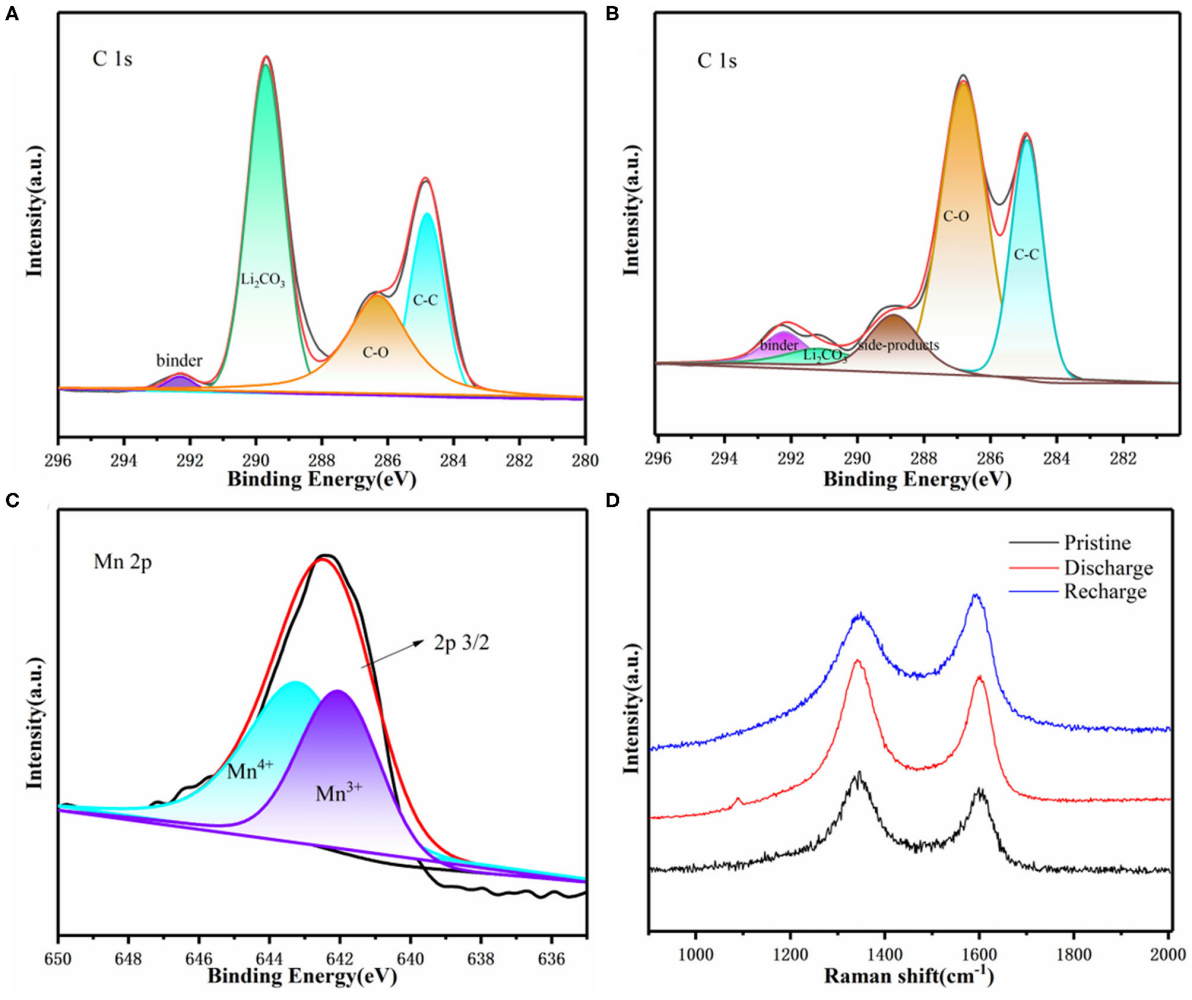
Li–CO_2_ battery **(A)** C1s XPS after first discharge, **(B)** C1s XPS after first charge, **(C)** XPS of Mn 2p_3/2_ after first charge. **(D)** Raman spectra of the pristine, first discharge and first charge.

We also used XPS analysis to explore related catalytic mechanisms (Figures 6A–C). Through the analysis of the C 1s spectrum of 3D KMO NWs, it was found that it corresponds to a high intensity Li_2_CO_3_ peak (289.8 eV) in the discharge state (Figure 6A). The peak almost disappeared during recharging, indicating that the 3D interconnected 3D KMO NWs nanowires have good catalytic performance for CO_2_ER (Figure 6C) (Xing et al., 2018). In addition, being similar to the original 3D KMO NWs, the high-resolution spectrum of Mn 2p_3/2_ also showed two constant peaks at 642.3 eV and 643.5 eV, which corresponding to Mn^3+^ and Mn^4+^, respectively, after recharging. The result shows that it is possible to suppress the Mn dissolution as the K^+^ ions steadily intercalated into the tunnels of 3D KMO NWs and bonded with the Mn polyhedrons, enhancing its inherent stability (Fang et al., 2019; Liu et al., 2019b). Therefore, it shows that 3D KMO NWs has good stability during charging and discharging. Through Raman spectroscopy (Figure 6D), the two peaks at 1,350 and 1,580 cm^−1^ are represented as the D-band and G-band of C, respectively. It is also found that there is a Li_2_CO_3_ peak at 1,080 cm^−1^ during discharging, and the peak disappears after charging, which further reveals the formation and decomposition of Li_2_CO_3_ during discharging and charging. The above results show that the product produced by Li-CO_2_ battery discharge is mainly Li_2_CO_3_, and Li_2_CO_3_ can be reversibly decomposed through the subsequent charging process.

## Conclusions

In summary, a K^+^ doped 3D KMO NWs catalyst for Li–CO_2_ batteries was provided and prepared by a simple hydrothermal method. It can effectively improve the performance of Li–CO_2_ batteries. This 3D network structure can promote Li^+^ diffusion, CO_2_ transfer and deposition/decomposition of Li_2_CO_3_. And the high content of K^+^ can stabilize the Mn-based positive electrode to improve the cycle stability. Compared with the lower K^+^ content of KMO and α-MnO_2_, the prepared 3D KMO NWs with high content of K^+^ have longer cycle life, lower overpotential and better rate performance, which is mainly due to the better morphology and high content of K^+^ that improves the ion conductivity and stabilizes the structure of the MnO_2_ hosts, thereby improving the catalytic performance of the MnO_2_ hosts. In addition, *ex-situ* XRD, SEM, XPS, and Raman analysis showed that the discharge product Li_2_CO_3_ was almost completely decomposed after recharging. Therefore, this strategy of doping K^+^ in MnO_2_ catalyst provides a new path for the further design of Li-CO_2_ battery.

## Data Availability Statement

The original contributions presented in the study are included in the article/Supplementary Material, further inquiries can be directed to the corresponding author.

## Author Contributions

ZT conducted the experiments and write the manuscript. MY and JL helped with experiments and data analysis. GZ and ZC explained the results. HZ, JD, and ZC supervised the research. All authors approved the submission of final manuscript.

## Conflict of Interest

The authors declare that the research was conducted in the absence of any commercial or financial relationships that could be construed as a potential conflict of interest.
