# Supplementary material for: Promoting the Performance of Li–CO2 Batteries via Constructing Three-Dimensional Interconnected K+ Doped MnO2 Nanowires Networks
Source: Front Chem. 2021 Apr 15;9:670612. doi: 10.3389/fchem.2021.670612 (PMC8082424; doi:10.3389/fchem.2021.670612)
Supplement: Supplementary file 1 [file Data_Sheet_1.docx]

Supplementary Material

Promoting the Performance of Li–CO_2_ Batteries via Constructing Three-Dimensional Interconnected K^+^ doped MnO_2_ Nanowires Networks

*Zhuolin Tang^1^, Mengming Yuan^1^, Huali Zhu^2^, Guang Zeng^1^, Jun Liu^1^, Junfei Duan^1^, Zhaoyong Chen^1*^*

*^1^ School of Materials Science and Engineering, Changsha University of Science and Technology, Changsha 410114, P.R. China.*

*^2^ School of Physics and Electronic Science, Changsha University of Science and Technology, Changsha 410114, P.R. China.*

*^*^ Corresponding author: E-mail address:* *[chenzhaoyongcioc@126.com](mailto:chenzhaoyongcioc@126.com)*


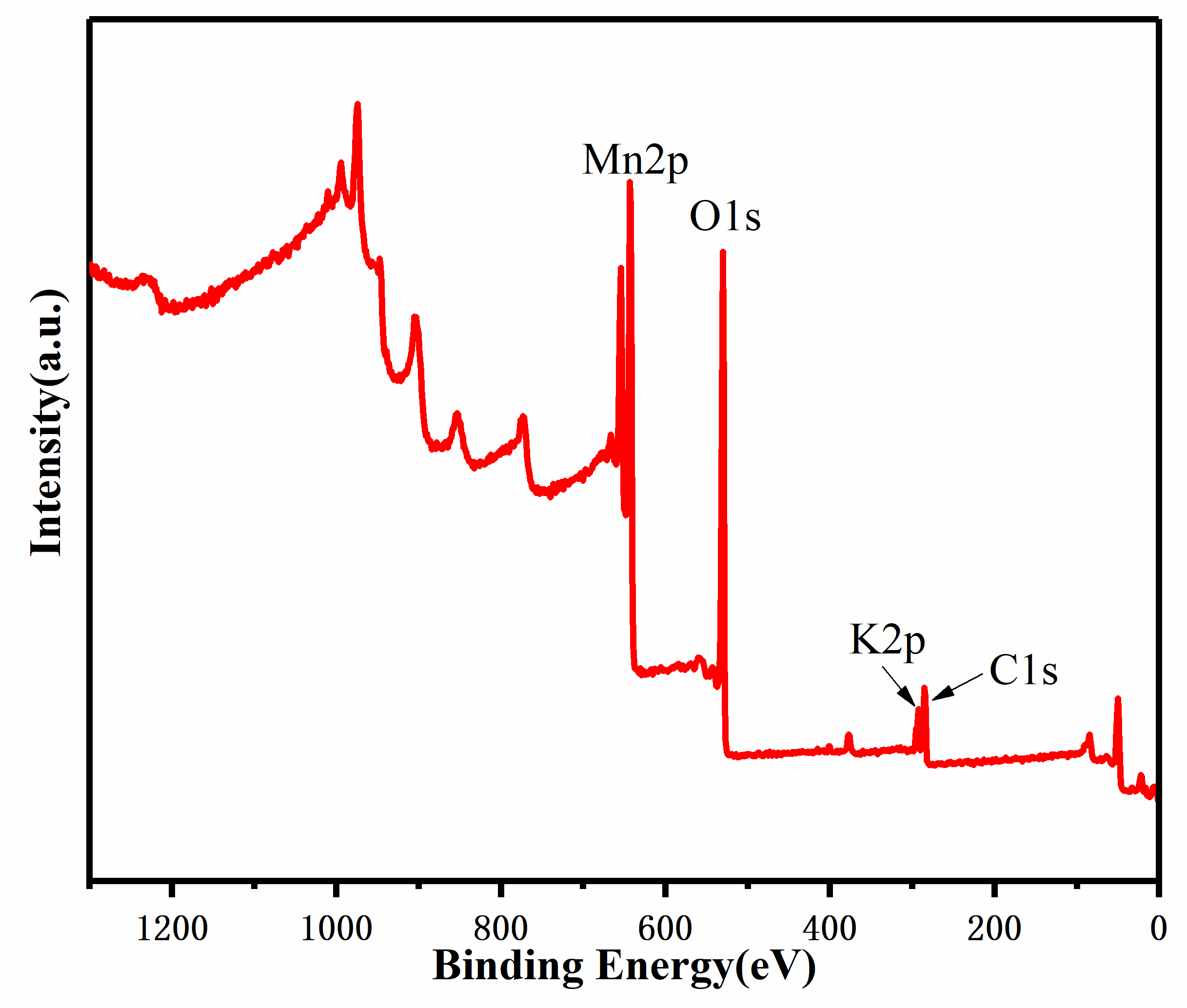


Figure S1. 3D KMO NWs XPS survey of full spectrum.


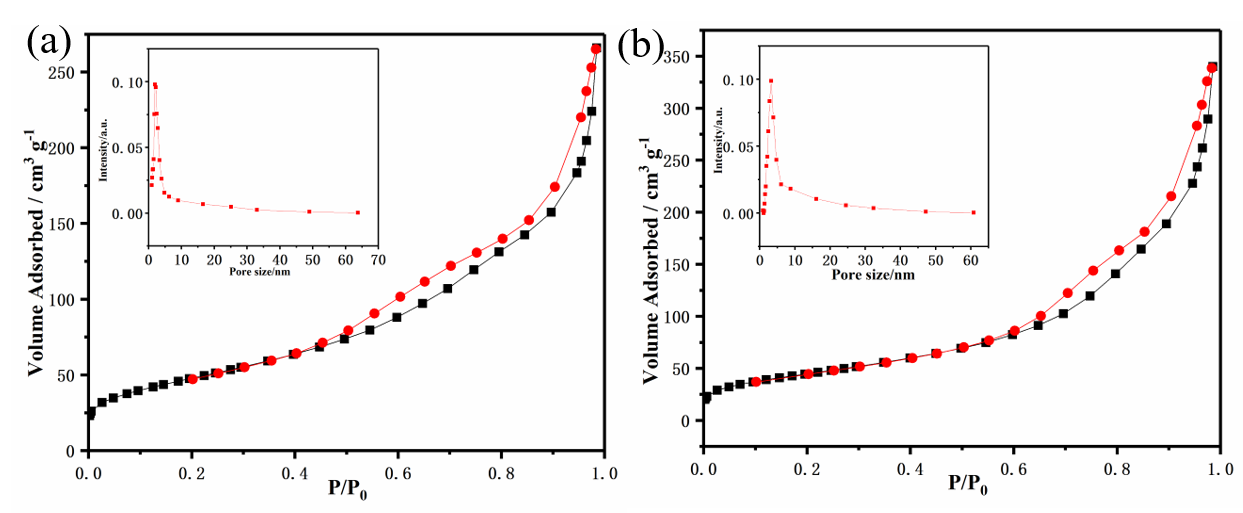


Figure S2. N_2_ adsorption/desorption isotherm curves of KMO (a) and α-MnO_2_ (b). The inset of (a-b) are the corresponding pore distribution of KMO and α-MnO_2_.


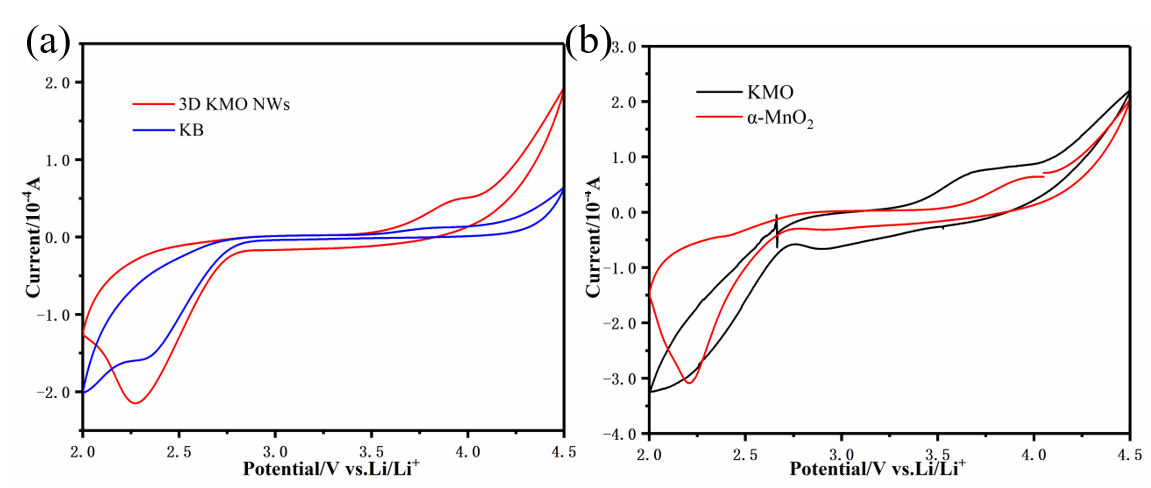


Figure S3 (a) CV curves of Li–CO_2_ batteries under an CO_2_ atmosphere with 3D KMO NWs and KB cathodes, (b) CV curves of Li–CO_2_ batteries under an CO_2_ atmosphere with KMO and α-MnO_2_ cathodes, respectively, at a scanning speed of 0.2 mV s^-1^.


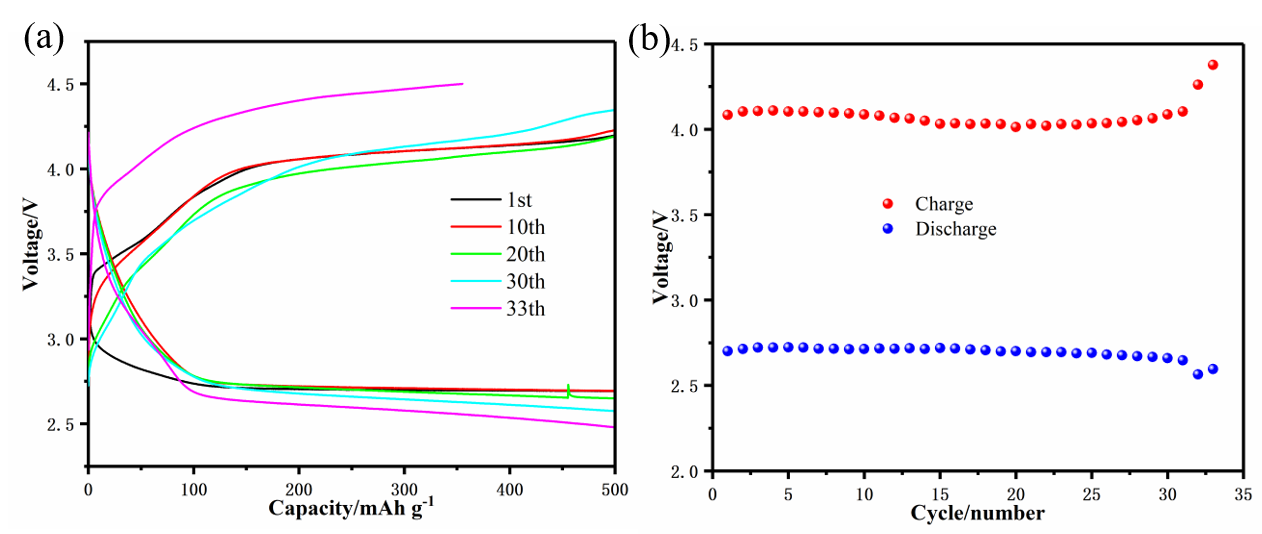


Figure S4. Electrochemical performance of KMO: (a) The discharge and charge and voltages upon cycling. (b) the relationship between the median voltage of KMO and the number of cycles.


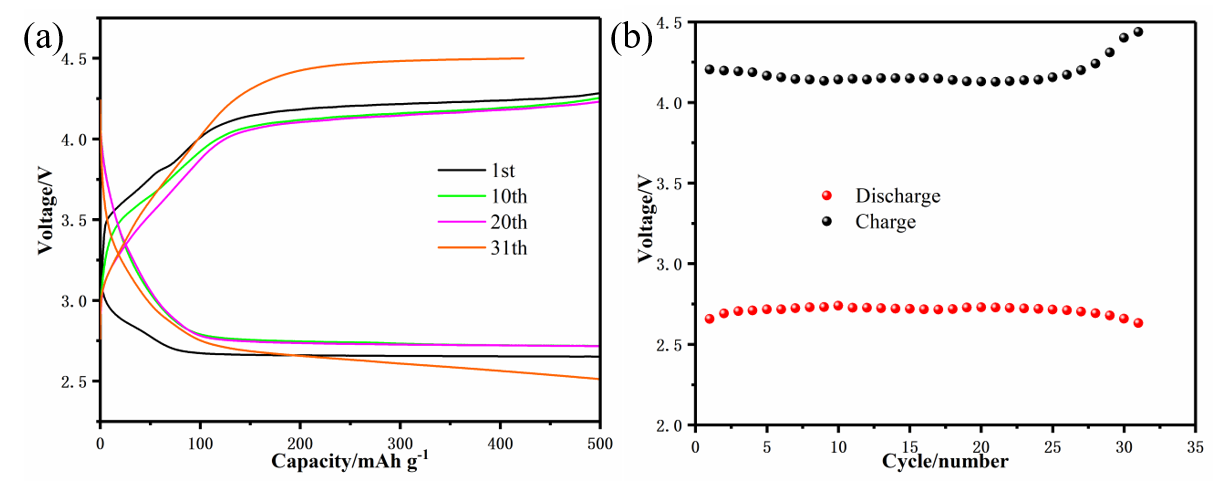


Figure S5. Electrochemical performance of α-MnO_2_: (a) The discharge and charge and voltages upon cycling. (b) the relationship between the median voltage of α-MnO_2_ and the number of cycles.


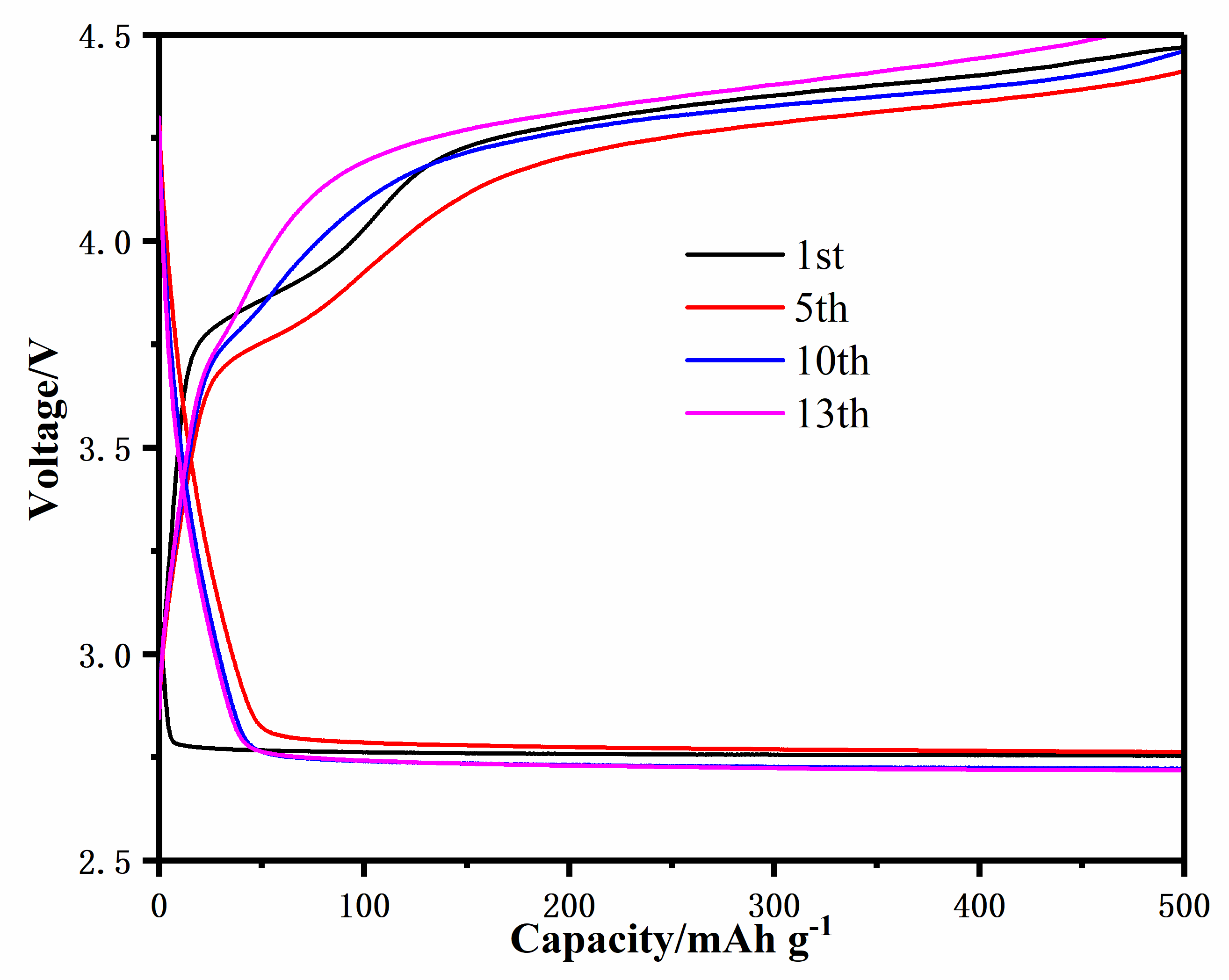


Figure S6. The discharge and charge and voltages upon cycling of commercial KB.
